# Body schema and body image as internal representations of the body, and their disorders. An historical review

**DOI:** 10.1111/jnp.12389

**Published:** 2024-09-08

**Authors:** Giuseppe Vallar

**Affiliations:** ^1^ Department of Psychology, and Mind and Behavior Technological Center – Mibtec University of Milano‐Bicocca Milan Italy

**Keywords:** asomatognosia, autotopagnosia, body image, body schema, body structural description, personal spatial neglect, phantom and supernumerary limbs

## Abstract

Since the early 1900s, the terms *body schema* and *body image* denoted the internal representations of the body. Bonnier's (1905, *Revue Neurologique*, 13, 605) schema is a conscious spatial representation of the size, shape, and position of the body, and of body parts, whose dysfunction brings about *aschématia*, and *hypo*‐, *hyper‐*, and *paraschématia*. The two schemata of Head and Holmes (1911, *Brain*, 34, 102) are an unconscious plastic postural schema, for the maintenance of posture and balance and for the coding of the position of body parts, and a conscious superficial schema, for the localisation of somatosensory stimuli. Pick's (1922, *Psychologische Forschung*, 1, 303) body schema refers to a structural description of the body, including the position of body parts and their spatial relationships, defective in autotopagnosia. Schilder's (1935, *The image and appearance of the human body*) body image is a comprehensive construct, covering physiological, evolutional, neurological and neuropsychological, psychiatric and sociological aspects. Lhermitte's (1939, *L'image de notre corps*) image, based on the views of the abovementioned authors, is defective in bodily neuropsychological disorders. The two terms have been used interchangeably, to denote (hemi‐)asomatognosia, anosognosia, autotopagnosia, depersonalisation, personal neglect, phantom and supernumerary limbs, somatoparaphrenia. Their properties have been summarized with general dichotomies: schema for action in space (“where” system), image for perception (“what” system), after primary sensory processing. While schema and image fractionated into multiple representations of aspects of the body, the two terms are still used to refer to some of these representations, and to their disorders.

## INTRODUCTION

This paper summarises and discusses the historical roots of the concept of the existence of one or more internal representations of the body, separate from the different sensory inputs that give rise to them. Current neurocognitive models emphasise the multisensory features of these representations (Berlucchi & Aglioti, 1997; Park & Blanke, 2019; Ronchi et al., 2018). Conversely, the first reports focused on the specific sensory inputs on which these representations were based: the vestibular system (Bonnier, 1905), the proprioceptive and the somatosensory systems, respectively supporting the building up of the unconscious postural and of the conscious superficial schemata of the body (Head & Holmes, 1911). Also Pick (1922) mentions the role of the different sensory modalities in the development of the body schemata, but he does not assign a prominent role to multisensory integration, since, in adults, they are mainly visual (see Poeck & Orgass, 1971, for review). The approach of Schilder (1935) is a comprehensive synthesis of Bonnier's (1905), Head and Holmes' (1911) and Pick's (1922) work. Some insight into Schilder's (1935) views comes from his account of the development of the body image, used as a synonimous of schema. According to Schilder (1935), ‘every sensation contributes to the building up of the body image’. ‘There is no fundamental difference between the various sensations in this respect’. However, ‘two factors, apparently, play a special part in the creation of the body image. The one is pain, the other the motor control over our limbs’ (p. 104). Schilder (1935) writes ‘And is the incomplete pain reaction a sign of the insufficient integration of the body‐image? We must study the libidinous structure of the body schema before we can try to give an answer to this question’. The main link between the early and the modern accounts appears then to be the moving of the focus from the individual sensory systems to the mechanisms of their integration, for the building up of the internal representations of the body.

## BEFORE THE “IMAGE AND THE SCHEMA”

The idea that an ‘internal representation of the body’ is stored in the brain was first put forward by the German physiologist Hermann Munk (1890), who took the view that the sensorimotor fronto‐parietal cortex (‘Fühlsphäre’) was the repository of images of movement. Carl Wernicke (1906) adopted Munk's ideas, in the context of his classification of psychiatric disorders (see Poeck & Orgass, 1971), emphasising the spatial aspect of body orientation, and the localisation of somatosensory perception. Sensory inputs give rise to images of body parts, stored in memory. These images constitute the ‘consciousness of the body’ or *somatopsyche*, distinguished from the ‘consciousness of the external world’. The psychiatric counterparts are *auto‐ or somato‐psychoses*, and *allopsychoses*. A patient with a disordered representation of body parts was described by Deny and Camus (1905). Deny was a medical doctor of the Salpétriére, the hospital where Jean Martin Charcot had been Professor of Clinic of Nervous Diseases (Charcot, 1879). The female patient of Deny and Camus (1905) reported that ‘…I do not feel my limbs anymore, I do not feel my head, I do not feel my hair anymore. … I try to think, and I cannot represent myself’. The neurological examination was normal, except for a possible deficit of position sense (‘the exam of position sense was difficult, since the patient, when asked about the position of a body part, with eyes closed, immediately moved it, before being able to provide a correct response’). A psychiatric diagnosis, though non‐typical, was made: ‘a sort of aberrant hypochondria due to the loss of corporeal consciousness’. The disorder was selective: neither other neuropsychological deficits, nor delusions, were present, and intelligence was preserved. No psychometric tests were performed. However, the neurological examination was accurate, complete and was reported in detail. Specifically, Deny & Camus (1905, p. 464) pointed out, without wishing to insist, ‘the *complete absence* in our patient of any kind of *delusional ideas or concepts and the integrity of her intellectual faculties and insight*’ (some words in italics). A similar case (‘Afunktion der Somatopsyche’) had been reported by Foerster (1903): a woman had any longer the notion of herself, did not any longer feel her head, her eyes, her hand, which became transformed. Both Deny and Camus' (1905) and Foerster's (1903) patients were severely distressed by this disorder, that is currently discussed under the rubric of *depersonalisation* (Salami et al., 2020; Sierra & Berrios, 1997). Deny and Camus (1905) draw a distinction between a ‘specific or sensory peripheral system’ and an ‘organic or myo psychical central system’ concerning the sensation of muscular activity. Due to transcortical associations, sensations from the external world and their memory images are related to images of internal, mainly visceral, sensations. This is *cœnesthesis*, namely: the general feeling of the existence of one's body, and of inhabiting it, that arises from multiple stimuli from various bodily organs. Finally, both Deny and Camus (1905) and Foerster (1903) traced back this deficit to a neurological brain disorder.

## PIERRE BONNIER (1905). THE ‘ASCHÉMATIES’, AS DISORDERS OF THE SPATIAL REPRESENTATIONS OF THE BODY

**FIGURE 1:**
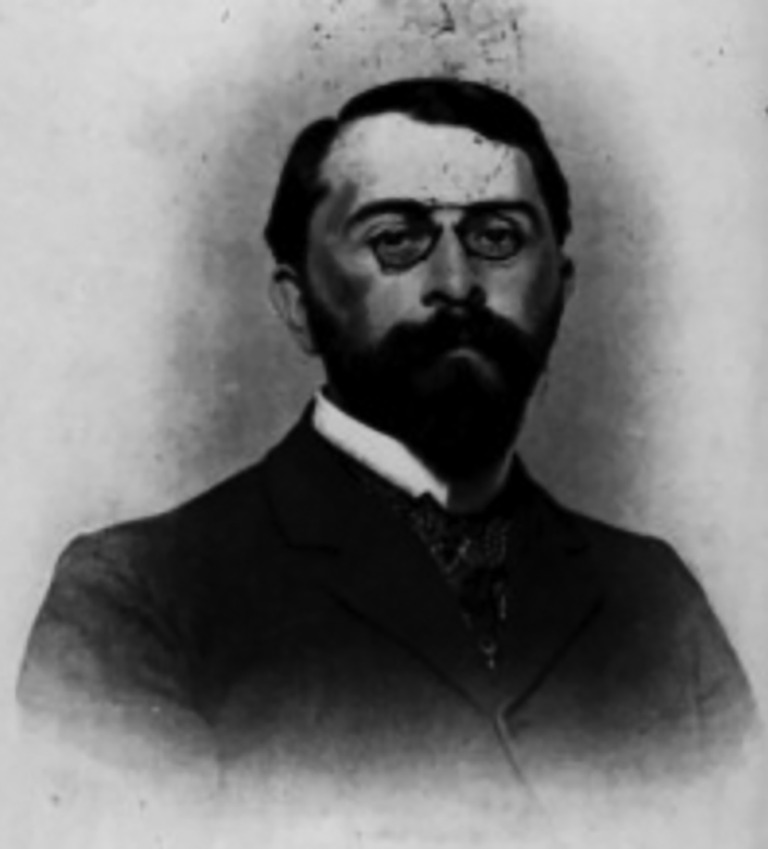
Pierre Bonnier (1861–1918). Credit: Vallar and Rode (2009), reprinted with permission of Elsevier.

Pierre Bonnier (Figure 1) was a French physician (Vallar & Rode, 2009), whose scientific interests concerned the auditory and the vestibular systems, and their disorders. Bonnier (1905) introduced the term *schéma*, to denote the internal spatial representation of the body, that he described as a topographical configuration, hence primarily spatial in nature. The schéma contains information about the volume of the body, and of body parts (‘… of schéma, topographic configuration, attitude. Because of this trouble some parts of ourselves cease to be represented in our body notion. When they take up too much room, there is *hyperschématie*; too little, *hyposchématie*; or a place which is not their right location, *paraschématie*. The *aschématia* is exactly an anaesthesia confined to the topographic notion, to the spatial representation, to the distribution, to the form, to the posture’).

Bonnier distinguished the schéma and the aschématie from the notion of cénesthésie and its pathological counterpart “*cénesthésiopathie*” (*cœnesthesiopathy*). Patients suffering from cœenesthesiopathy report the absence or decrease of the experience of their own body or of parts of it (e.g., head, organs, limbs), and a change of sensory experience (density, size, shape, spatial position). Body parts may be felt disproportionately longer, shorter, bigger, smaller, misshapen, displaced with respect to their spatial position. These subjective experiences about their own body, felt by patients as unusual and bizarre, are stressful. Bonnier (1905) dismissed the term ‘cénesthésiopathie’ (‘… because common sensations have no physiological significance; what all sensations have in common, as far as the question we are interested in is concerned, is the topographic definition, the notion of space, of localisation. … I then assigned primacy to the spatial notion, and I termed spatial sense all kinds of sensitivity, both peripheral and central, contributing to define objective and subjective orientation, the latter being the perception of our position with respect to environmental objects, objectively oriented, of our attitude, and of our changes of position and attitude’). Bonnier mentioned Wernicke's distinction between ‘the specific sensory element’, and ‘a myopsychic element’, concerned with the sensation of muscular activity and movement. Bonnier then noted that the process of spatial localisation applies to the different sensory modalities, providing a common medium.

Bonnier described several cases showing disorders of the schéma. All patients suffered from peripheral vestibular disorders, with no mention of any neurological deficit. Bonnier reported only subjective symptoms, but not, at variance from Deny and Camus (1905), the results of a neurological examination. Three impairments were clinically described. (i) *Aschématia*. For example, patient #a showed a complete and selective inability to localise sensory stimuli in different modalities, even though they were adequately perceived, and a global disorientation of the body. The patient reported ‘In these short moments of the attack… I continued to feel everything, but nothing is no longer anywhere, and I too am no longer anywhere’. Patient #b reported a ‘total suspension of any idea of his personality’ and ‘very carefully noted the departure and return of his identity’, a symptom that could be described as absence or *petit mal* seizures, although he was able to report it. (ii) *Hyperschématia*. Patient #g ‘felt his head becoming enormous, immense, lost in the air; his body disappeared, and all his being was reduced to his face’. (iii) *Paraschématia*. A patient felt that ‘his heart and the left side of the chest moved away from their normal site and vanished falling on the left’. The novel elements of Bonnier's report may be summarised in two points. (i) There is a conscious spatial internal representation of the body (schéma), concerned with the orientation of the body, and of body parts, their size and shape, and the localisation of sensory inputs, underpinning the very notion of corporeal being. The sensory image, which supports the perception of a certain modality (sound, light, warmth, etc.), is distinct from the body schéma. (ii) Peripheral deficits of the vestibular system, found in all patients, may bring about disorders of the body schema. Hécaen (1972), mentioned Bonnier (1905) as a precursor of the concept of an internal representation of the body, and his view that the vestibular system contributes to the building up of the body schema. This suggestion was supported by the finding that asymmetrical vestibular stimulation improves not only extra‐personal neglect, but also its bodily manifestations, such as *somatoparaphrenia* (typically, a delusion of disownership of contralateral body parts, most frequently left‐sided, Vallar & Ronchi, 2009), *personal neglect*, and *anosognosia for hemiplegia* (Bisiach et al., 1991; Cappa et al., 1987). However, Hècaen also noted the absence of neurological correlates of the body schema.

## HENRY HEAD, GORDON HOLMES AND BRITISH NEUROLOGY AND PSYCHOLOGY

**FIGURE 2:**
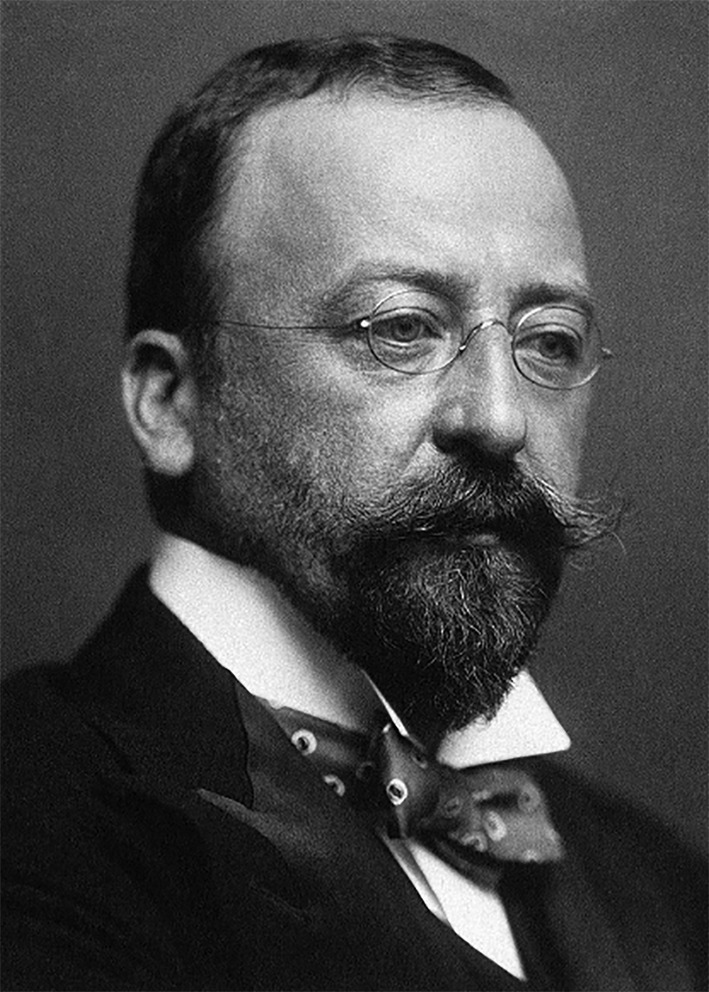
Sir Henry Head (1861–1940). Credit: Formal portrait of Sir Henry Head looking right. https://wellcomecollection.org/.

**FIGURE 3:**
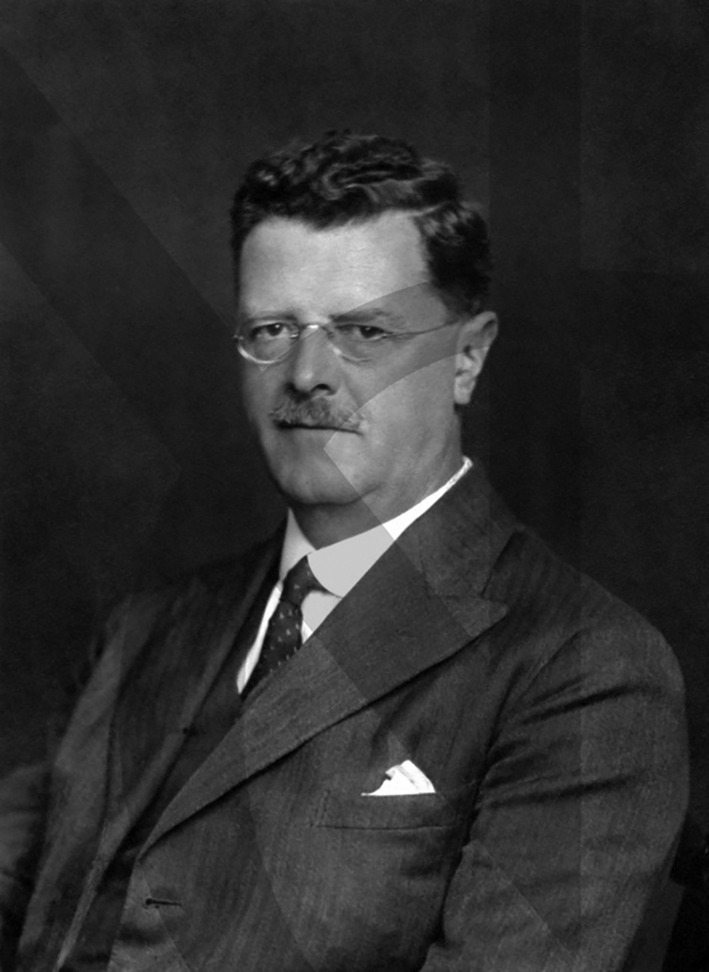
Sir Gordon Holmes (1876–1965). Credit: National Portrait Picture Gallery, http://www.npg.org.uk/collections/search/portraitLarge/mw109878/Sir‐Gordon‐Morgan‐Holmes, reprinted with permission.

The term schema was used by the British neurologists Henry Head and Gordon Holmes (Figures 2 and 3) in their influential paper *Sensory disturbances from cerebral lesions* (Head & Holmes, 1911, p. 186 ff.). ‘For this combined standard, against which all subsequent changes of posture are measured before they enter consciousness, we propose the word “schema”’. The term image refers to past impressions that ‘rise into consciousness’. ‘By means of perpetual alterations in position we are always building up a postural model of ourselves which constantly changes. Every new posture or movement is recorded on this plastic schema, and the activity of the cortex brings every fresh group of sensations evoked by altered posture into relation with it. Immediate postural recognition follows as soon as the relation is complete. … In the same way, recognition of the locality of the stimulated spot demands the reference to another “schema.” For a patient may be able to name correctly, and indicate on a diagram or on another person's hand, the exact position of the spot touched or pricked, and yet be ignorant of the position in space of the limb upon which it lies… This faculty of localization is evidently associated with the existence of another schema or model of the surface of our bodies which also can be destroyed by a cortical lesion. The patient then complains that he has no idea where he has been touched. He knows that a contact has occurred, but he cannot tell where it has taken place on the surface of the affected part’. Conversely, patient Hn. (Case 14) ‘never failed to localize the stimulated spot correctly, although he could not tell the position of his hand’. Head & Holmes (1911, p. 188) also suggested that the body schema could extend to peri‐personal space. ‘Anything which participates in the conscious movement of our bodies is added to the model of ourselves and becomes part of these schemata: a woman's power of localization may extend to the feather in her hat’. Similarly, ‘the body‐image of a motorist, an air‐pilot, an equestrian temporarily includes part at least of the automobile, the aircraft, or the horse. A surgeon with his probe, a blind man with his white stick for the time being are endowed with an extension of corporeal awareness’ (Critchley, 1979, p. 94).

Head and Holmes' (1911) conclusions were based on a detailed neurological exam, with some quantitative assessment of tactile function (e.g., von Frey's hairs). Bonnier's (1905) and Wernicke's papers were not cited. The work of Munk (1890), who regarded the cortex as ‘the repository of images of movement’ (p. 186; see also Critchley, 1953, p. 108), was discussed.

## ARNOLD PICK

**FIGURE 4:**
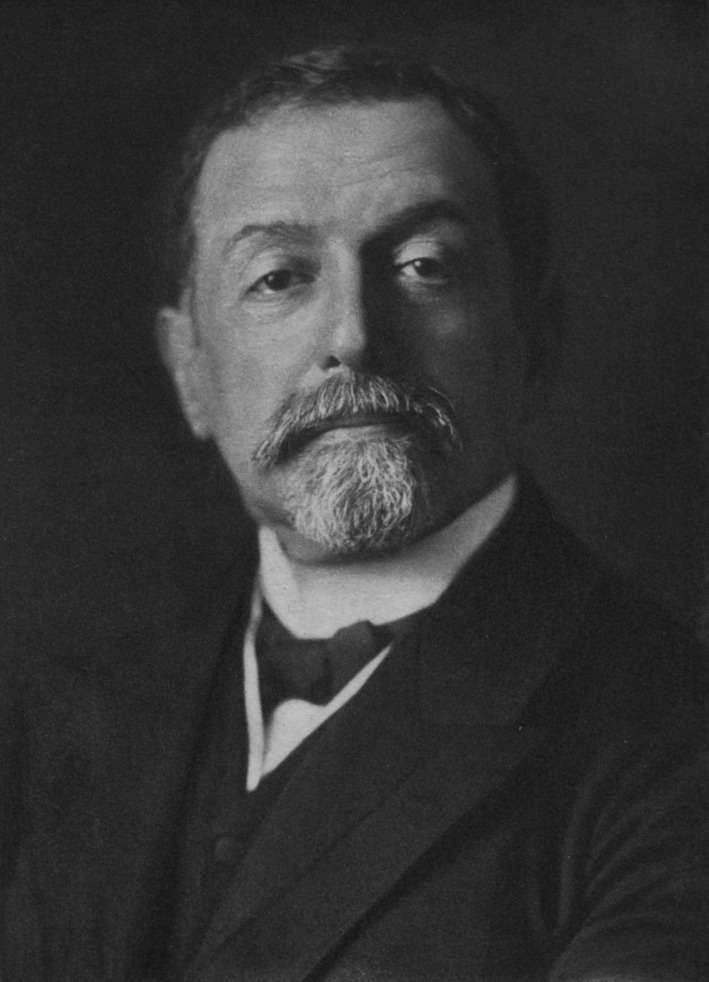
Arnold Pick (1851–1924). Credit: Wikimedia Commons, https://commons.wikimedia.org/wiki/File:Arnold_Pick_(1851‐1924).JPG.

Arnold Pick (Figure 4) was a neuropsychiatrist, professor in Prague. Pick's views on the body schema were developed between 1908 and 1922, and based on clinical observations, with reference to the ideas of Munk and Wernicke (see Poeck & Orgass, 1971; Semenza & Delazer, 2003). Using Head and Holmes' (1911) terminology, Pick suggested the existence of several schemata (Conrad, 1933; Poeck & Orgass, 1971), for the different sensory modalities and for different body parts, with the schemata concerned with the representation of the body surface being particularly relevant. Consciousness of one's own body (‘Bewußtsein der Körperlichkeit’) is based on these schemata, with an important role of the visual image (‘Optisches Vorstellungsbild’). In Pick's view, in children the representation of the corporeal self is mainly based on tactile and kinaesthetic sensation; the role of visual sensations becomes more and more relevant, and, in adults, body representations (images) are mainly visual. This primacy of visual sensations may be related to the important role of the representations of the body surface.

## PUTATIVE NEUROPSYCHOLOGICAL IMPAIRMENTS OF THE BODY SCHEMA/IMAGE

Pick's main empirical contribution to the concept of body schema was the description, in two patients with dementia, and in one patient with right hemiparesis, of *autotopagnosia*, namely: a deficit in the localisation by pointing to and touching body parts on the patient's own and on another person's body, on verbal command. The patients' ability to recognise and name their own body parts, singled out by the examiner, was spared. The selectivity of Pick's observations has been called into question (Denburg & Tranel, 2003; Poeck & Orgass, 1971), since patients with a putative autotopagnosia often show associated deficits, potentially responsible for autotopagnosia, most frequently aphasia, but also apraxia, reaching disturbances, visuo‐spatial deficits, and general cognitive impairment. More specifically, De Renzi and Scotti (1970) reported the case of a left brain‐damaged patient who was impaired in pointing to body parts on verbal command, although he was perfectly able to name the same parts when the examiner pointed to them. However, the same dissociation between pointing and naming was found with parts of objects, as a bicycle. De Renzi and Scotti (1970) concluded then that the patient's basic disturbance was the inability to analyse a whole into its parts, rather than a selective deficit of the body schema or image. However, a few single case studies of patients with a ‘pure’ autotopagnosia, with no associated deficits, that may account for the disorder, have been reported. In patient J.P.B., with a left hemisphere metastatic tumour, Ogden (1985) found a dissociation between defective pointing to his own, the examiner's, and a doll's body parts, and a preserved localisation by pointing of parts of objects, plants and animals, and of items of clothing on his body. Naming body parts and parts of objects and animals was also preserved. In patient D.L.S., suffering from Alzheimer disease, Sirigu et al. (1991) found a defective pointing to her own (more severe impairment) and to the examiner's body parts, both on verbal command and in a non‐verbal condition. Conversely, naming of body parts and localising both different parts of objects and targets attached on body parts were preserved. In addition to autotopoagnosia, Pick interpreted also phantom limb phenomena, and the inability to appreciate the position of body parts as deficits of the body schema.

In the 1940s, the concepts of body schema and of body image were used, by the British neurologists Russell Brain (body schema, 1941), and Martin Roth (body image, 1949), to interpret the bodily components of the neglect syndrome, typically associated with damage to the right hemisphere (Vallar et al., 2024). In the description of the deficits of two right brain‐damaged patients, Roth (1949) used the term body image as a synonymous of body schema, and traced back the origin of the concept to Pick, and Head and Holmes (1911).

Macdonald Critchley, in the book *The Parietal Lobes* (1953), proposed a taxonomy of the disorders of the body image, regarded, for the time being, as synonymous with the body schema. Critchley did not refer to Head and Holmes' (1911) concept of body schema. This was discussed in chapter IV: ‘Disorders of tactile function’.

Several unilateral deficits were listed as ‘Disorders of the body image’ (chapter VIII).

- *Unilateral spatial neglect* (motor neglect: a poverty of movements and deliberate willed action of the contralateral limbs, without hemiparesis).
- *Tactile and visual neglect or inattention*, as revealed by the technique of double simultaneous stimulation, that brings about a failure to report contralateral stimuli (sensory extinction). Inattention to contralateral visual stimuli (presented in extra‐personal space) might be considered a deficit of the body image, since it is produced by the simultaneous activation of distance (from the body) receptors.
- Five deficits of awareness of hemiparesis contralateral to the side of the lesion: (a) *Anosodiaphoria* (lack of concern over the existence of hemiparesis); (b) *anosognosia* (unawareness of hemiparesis); (c) *defective appreciation of the existence of hemiparesis with rationalisation*; (d) *denial of hemiparesis*; (e) *denial of hemiparesis, with confabulation*.
- *Hemi‐asomatognosia, hemi‐depersonalisation* (loss of awareness of one body‐half, which may or may be not paralysed). According to Critchley (1953), in hemi‐asomatognosia the patient's ‘feeling is one *as if* no left arm or leg existed’. … This feeling of “nothingness” is most often spoken of as asomatognosia or hemi‐asomatognosia”’ (p. 237). Schilder (1935, pp. 28, 36) called this disorder ‘body image imperception’, which could involve one half of the body. Ehrenwald (1931) had used the term ‘depersonalisation’, which again could involve one half of the body. A distinction was later drawn between ‘conscious’ and ‘nonconscious’ hemi‐asomatognosia (Frederiks, 1985, pp. 374‐375). Patients with a conscious deficit typically complain that one side of the body, or part of it, contralateral to the side of the hemispheric lesion, has disappeared, and they are unable to locate it (e.g., Roth, 1949, p. 91, ‘she was often unable to find her hand and, on one occasion, she was observed searching for it’). Patients may also show a para‐schematic type of disorder, ‘He complained that he did not know where the left side of his body was. … He said that the left side of his body felt different from the right side and as if it were on the right side’ (Brain, 1941, p. 258). Conscious asomatognosia has been distinguished from somatoparaphrenia, in which frank delusional beliefs are present (Vallar & Ronchi, 2009). Such delusions are often simple, as disownership (‘hand belonging to the doctor’), sometimes more complex, for example: the left side was felt different from the right side, recognised as ‘self’ and ‘good’: The left side was evil, controlled by external agents (the Devil, the patient's deceased father) attempting at inducing the patient to perform evil acts (Nightingale, 1982, p. 464). Patients with left hemiplegia and anosognosia may develop symptoms resembling depersonalisation or Cotard's Syndrome (the delusional belief that one is dead or non‐existent, Dieguez, 2018). A patient with a left hemiplegia complained of his left arm as ‘being alien (“fremd”, unfamiliar), dead, gone away; he felt a strange hand bigger than the previous one’ (Ehrenwald, quoted by Sierra & Berrios, 1997, p. 221), showing hyperschematia. Patients may show *misoplegia*, namely: hatred of contralateral body parts, ranging from manifestations restricted to verbal aggression towards a limb, to physical acts such as striking and beating the hemiplegic extremity (Loetscher et al., 2006). These descriptive examples suggest that there may be a continuum between conscious asomatognosia, somatoparaphrenia and misoplegia. Nonconscious hemi‐asomatognosia is a synonymous of the currently more used term ‘personal neglect’, namely: a deficit in reaching and exploring the contralateral side of the body, or of body parts, such as the hand (Bisiach, Perani, et al., 1986), and in activities as combing, shaving or making up, that require the planning and execution of willed actions in both sides of the face. For example, ‘A female patient was found to have applied cosmetics to one side only of her face and lips, and to have left her hair unkempt, with kirby grips *in situ*, except on the right side, where she was soignée and neat’. (Critchley, 1953, pp. 227–228). A distinction may be drawn between representations involved in the monitoring of the function of motor (output) systems and representations concerned with awareness and ownership of the body. In the series of Bisiach, Vallar, et al. (1986), five patients showed anosognosia for left hemiplegia without left personal neglect, assessed by requiring patients to reach their left hand with a leftward movement of the right arm. Right brain‐damaged patients with contralateral (left) somatoparaphrenia (Vallar & Ronchi, 2009) may show anosognosia for left hemiplegia without left personal neglect/nonconscious hemi‐asomatognosia (patient A.R., Bisiach et al., 1991). In patient G. H. with left somatoparaphrenia, the opposite dissociation was found, namely: left personal neglect with no anosognosia for left hemiplegia (Halligan et al., 1995). Similarly, during right intracarotid barbiturate infusion, patients with anosognosia for left hemiplegia may exhibit or not defective ownership of the left hand (somatoparaphrenia), assessed by an identification task (Adair et al., 1995), not requiring any leftward movement, and then ruling out any interpretation in terms of leftward directional hypokinesia (Bottini et al., 1992). Lesion‐based evidence from right brain‐damaged patients provides some support to the view that independent bodily representations subserve the monitoring of motor function and the sense of body awareness and ownership. Anatomo‐clinical correlation studies appear to indicate a not complete overlap of networks, whose damage is associated with these disorders: they include primarily the frontal, temporal, parietal, and insular associative cortices (Yeo et al., 2015), and then the sensorimotor cortex, the underlying white matter fibre bundles, and subcortical nuclei, (Berlucchi & Aglioti, 2010; Berti et al., 2005; Committeri et al., 2018; Gandola et al., 2012; Moro et al., 2016; Pia et al., 2004; Vocat et al., 2010).

In sum, these unilateral deficits, contralateral to the side of the hemispheric lesion, and components of the syndrome of unilateral spatial neglect (Hécaen & De Ajuriaguerra, 1952; Vallar et al., 2024; Veronelli & Vallar, 2024) are typically more frequent, severe, or both, after damage to the right hemisphere.

v *Undue heaviness, deadness or lifelessness of one half*. Bonnier's terminology was used to classify the different types of distortion from parietal disease, or a psychotic type of illness. Critchley pointed out that aschematia (total asomatognosia or depersonalisation), as well as segmental depersonalisation, are frequently associated with psychiatric disorders. The complaint that the affected limb is unduly heavy or swollen may be considered a manifestation of hyperschematia (Critchley, 1953, pp. 240–241). These hallucinations (Denes, 1989) are usually paroxysmal, and may occur during attacks of migraine (e.g., Lippman, 1952; Podoll & Robinson, 2000) or epilepsy (see also patient #b of Bonnier, 1905), with a focus in the parietal lobe (e.g., Salanova et al., 1995; Todd, 1955), and after focal brain damage (e.g., Rode et al., 2012).
vi *Phantom third limb*, *associated with hemiparesis*.

Two other deficits, interpreted in terms of a defective body image or schema, are not tabulated by Critchley (1953).

vii *Sensory (tactile) allæsthesia or allochiria*. ‘Allæsthesia was observed, painful stimuli to the left upper limb being referred to the right’ (Roth, 1949, p. 91). ‘Sensory stimuli applied to the left half of the body were sometimes referred to the right’ (Brain, 1941, p. 264). These terms denote the pathological phenomenon whereby sensory stimulations, delivered to the contralateral side of the body (tactile stimuli) or presented in the contralateral visual half‐field or half‐space (visual and auditory stimuli), are consciously perceived, but referred to the symmetrical position in the ipsilateral side. Allæsthesia is frequently associated with right brain damage, involving then a left‐to‐right displacement (Stone & Vermeulen, 2016).
viii *Apraxia for dressing*. Patients reported by Brain (1941) and by Roth (1949) showed this type of apraxia, independent of other apraxias. Brain (1941 p. 266) concluded that ‘Apraxia for dressing is also independent of constructional apraxia, though clearly a very similar disorder. It appears closely related to the disorder of the body scheme for one half of the body, for the recognition of the structure of clothes must necessarily be intimately related to the perception of the body. Hence, a lesion in either hemisphere which disorganizes the body scheme may cause apraxia for dressing’. According to Critchley (1953, pp. 159–160) one of the several causes of a disorder of the art of dressing is contralateral personal neglect, ‘resulting from a disordered body image’. Dressing apraxia has been regarded not as a discrete deficit, with specific pathological mechanisms, but as due to spatial neglect, other visuospatial deficits or both (Heilman et al., 2003, p. 305). The disorder has been sporadically reported in clinical case studies (a patient with an infarction in the left parietal lobe, with no other apraxias and spatial neglect, Yamazaki et al., 2001).

Three deficits are bilateral, associated with damage to the left hemisphere, or diffuse cortical atrophy.

- *Autotopagnosia* is associated with diffuse cortical atrophy (dementia), as in Pick's Cases # 1 and #3, and with left hemispheric damage, as in Case #2 (see tab. 15.1, p. 226, of the review of Semenza & Delazer, 2003). These early correlations have been subsequently confirmed in two patients by Ogden (1985, left parietal lesion) and by Sirigu et al. (1991, diffuse cortical atrophy).
- *Finger agnosia* is the patients' inability to recognise, indicate on request, name, or choose with open eyes, individual fingers, either of their own hands or of the hands of other persons. According to Gerstmann (1940, p. 400), ‘in finger agnosia the so‐called body scheme, the postural model of the body—that is, the image which a person normally has of his body and the spatial relation of its parts were affected by a focal cerebral lesion in one sphere only, and, indeed, the most significant, differentiated and vulnerable one—the sphere concerned with the individual fingers’.
- *Right–left disorientation* in respect to the patient's own body, as well as that of other persons, is the patients' tendency to confuse the left and right halves of their body, and of symmetric parts of another person's body. This symptom was reported by Gerstmann (1940), as a component, together with finger agnosia, acalculia and agraphia, of Gerstmann's syndrome, whose neuropathological correlates are a lesion of the posterior parieto‐occipital cortex of the left hemisphere (Rusconi, 2018; see Kleinschmidt & Rusconi, 2011, for a discussion of Gertsmann's syndrome as a disconnection syndrome). Finally, it is worth noting that, with reference to finger agnosia and right–left disorientation, also Gerstmann (1940, p. 406) used the two terms (schema and image) interchangeably.

Hécaen and De Ajuriaguerra (1952) consider *asymboly for pain* as a disorder of the body schema/image associated with left brain damage. Patients with asymbolia for pain can recognise pain but lack appropriate motor and emotional responses to painful stimuli applied anywhere on the body surface (Berthier et al., 1988).

## PAUL SCHILDER

**FIGURE 5:**
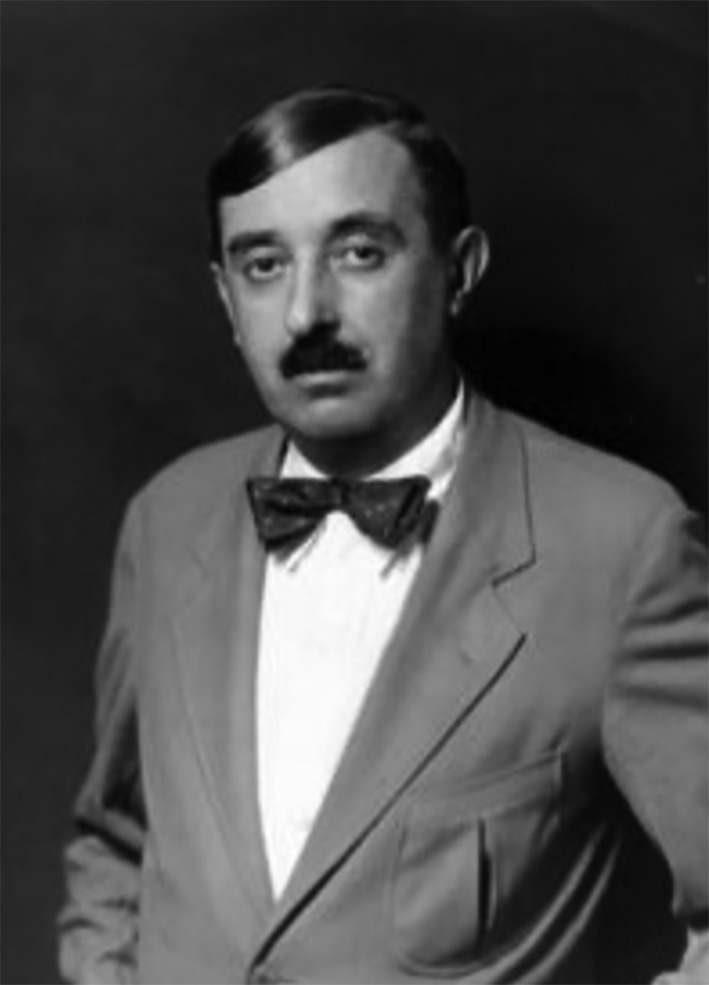
Paul Schilder (1886–1940). Credit: Wikimedia Commons, https://it.m.wikipedia.org/wiki/File:Paul_Schilder.jpg.

Paul Schilder (Figure 5) is nowadays remembered predominantly for his contributions to psychiatry and psychotherapy. Schilder was also a neurologist, who, in his early years, published on neuropathological topics. Schilder summarised his work about the internal representations of the body in the book *The image and appearance of the human body* (1935), discussing the body image in its neurophysiological, neuropsychological, psychoanalytic and psychiatric, and sociological aspects. Schilder (1923) had previously published a small book titled *Das Körperschema: Ein Beitrag Zur Lehre Vom Bewußtsein Des Eigenen Körpers*, in which he interpreted allœsthesia and allochiria, the phantom limb, autotopagnosia and its relationships with apraxia, as deficits of the body schema. The tables of content of the 1923 and of the 1935 books show some overlap between the two concepts, although the image covers a wider range of processes and deficits. Lhermitte (1951, p. 434) approved this change of the title ‘because it is not a body scheme we possess, but more exactly an image of our body’. One reason for this title change could have been that the term image may readily cover also visual phenomena related to the body. In a somewhat similar vein, Frederiks (1985) draw a distinction between ‘body schema’ and ‘body experience’ . The term ‘schema’ is used for the awareness of the spatial characteristics of one’s own body, and is formed by current and previous sensory information. This account is largely based on the views of Bonnier (1905) and Head and Holmes (1911). The term ‘experience’ is more comprehensive, including psychological, situational, emotional and intentional factors. According to Frederiks (1985), the term ‘schema’ is mainly used in neurology and neuropsychology, the term ‘experience’ in psychology and psychopathology.

Schilder (1935) confirmed Bonnier's (1905) suggestion of an association between disorders of the body schema and vestibular dysfunctions, reporting several clinical observations of patients whose visual and somatosensory phenomenal experiences were modulated by the activity of the vestibular system. ‘One of the patients of Hoff and myself felt her neck swell during dizziness. The same patient felt that her extremities had become larger’ (Schilder, 1935, p. 117). Autotopagnosia, imperception of the impairment of somatic functions (anosognosia for hemiplegia) and of the body or of parts of it (asomatognosia), left–right disorientation, phantom limbs, and finger agnosia are disorders of the body image. Overall, Schilder discussed all neurological and psychiatric disorders concerning the body as dysfunctions of the image.

## JEAN LHERMITTE AND FRENCH NEUROLOGY

The French neurologist Jean Lhermitte summarised his views about the body image in the book *L'image de notre corps* (1939). Lhermitte's eclectic approach was largely based on the suggestions by Bonnier (1905), Head and Holmes (1911), and Pick (1922). According to Lhermitte, the image of one's own physical character is provided by various tactile, vestibular, kinaesthetic, and visual sensations and perceptions, with proprioception and vision interacting with each other to maintain a perception of reality. The body image is a synthesis of perceptions of extero‐, intero‐, and proprioceptive excitations, and of representations, namely: memories, continuously updated. Through the perspective of the body image, Lhermitte and Tchehrazi (1937) approached the neurological accounts of phantom limb phenomena, and of disorders typically contralateral to the side of the lesion, more frequently involving the left side of the body. These included: anosognosia of hemiplegia, hemi‐asomatognosia, somatoparaphrenia (Vallar & Ronchi, 2009). *Autoscopy* ‘is a visual experience where the person sees an image of him/herself in external space, viewed from within his/her own physical body (Dening & Berrios, 1994, p. 108)’, ‘is a sensation the patient has of seeing his body image as reflected in a mirror’ (Lhermitte, 1951 p. 431). For example, ‘…our patient had the impression that his body had split in two and that this double was lying very close beside him in a state of imminent death’ (Lhermitte, 1951 p. 431). *Heautoscopy* has been defined as the emotional encounter with a ‘phantom double’ of oneself, who moves independently and makes the person wonder whether the observer's perspective is centred on the body or on the phantom (Brugger & Lenggenhager, 2014). Limb apraxia may also be interpreted in terms of a deficit of the body image, since the organisation of action presupposes the existence of an internal spatial (visuo‐tactile) representation of the body. A complete or partial dissolution of the body image may be a major pathological mechanisms of apraxia (Lhermitte & Tchehrazi, 1937). A more selective deficit of the body image is finger agnosia (Gerstmann, 1940). Finally, autotopagnosia is too considered a deficit of the body image. Lhermitte used the terms schema and image interchangeably, for instance when finger agnosia is described (Lhermitte & Tchehrazi, 1937, p. 20).

## THE HERITAGE OF THE BODY SCHEMA AND OF THE BODY IMAGE IN MODERN NEUROPSYCHOLOGY

The concepts of body schema and of body image have been influential, and widely used terms, in the interpretation of neuropsychological and psychiatric disorders concerning the body. A PubMed search (May 30th, 2024) displays over 5.900 results for each term. A journal titled *Body Image* is currently published, dealing with several not neuropsychological topics, as body appreciation and esteem along the life span, body shape, muscularity, eating disorders. The main features of the body schema and image, according to Bonnier (1905), Head and Holmes (1911), Pick (1922), Schilder (1935) and Lhermitte (1939) are summarised in Table 1. In three accounts, there is a prevailing sensory modality on which the schema is built up: vestibular (Bonnier), somatosensory, including proprioceptive, tactile, and painful inputs (Head & Holmes), visual image, in adults (Pick). Schilder's (1935) and Lhermitte's (1939) descriptions have multisensory features. Also in modern models emphasis is given to the multisensory integration of the diverse sensory inputs, and of motor feedbacks (Limanowski, 2022; Maravita, 2006).

**TABLE 1:** Functional features of the body schema and of the body image, according to the physicians and neurologists who first introduced and used the two terms.

Author	Bonnier (1905)	Head and Holmes (1911)	Pick (1922)	Schilder (1935)	Lhermitte (1939)	Modern models
Name	Schema	Schema	Schema (visual image)	Image	Image (*L'image de notre corps*)	Sirigu et al. (1991, autotopagnosia) Schwoebel & Coslett (2005, brain‐damaged patients) Longo et al. (2010, brain‐damaged patients, healthy participants, neuroimaging, neurophysiology) de Vignemont (2010, deficits of bodily awareness) Moseley et al. (2012, illusions of body ownership)
Sensory input	Vestibular (mainly)	Somatosensory Proprioceptive Touch Pain	Multisensory (mainly visual)	Multisensory	Multisensory	
Representation	*Conscious spatial* (size shape, orientation, position of whole body, of body parts)	*Unconscious plastic postural schema* (balance, posture, position of body parts) *Conscious superficial schema* (localisation of somatosensory stimuli on the body surface) Extension of the scheme to include objects (e.g., tools) in near peripersonal space	Whole body, position of body parts in the body	Includes Bonnier's, Pick's and Head & Holmes' schemata. Evolutional, physiological, neurological, psychiatric and psychoanalytic, sociological aspects of the image	Includes Bonnier's, Pick's and Head & Holmes' schemata, and Schilder's image	
Bodily deficits	*Áschematie* *Hyperschématie* *Hyposchématie* *Paraschématie*	Defective *Perception of body and of body parts' spatial position* *Localisation of somatosensory stimuli on the body surface*	*Autotopagnosia*	Neuropsychological *(Hemi)Asomatognosia* *Conscious* *Nonconscious (Personal neglect)* *Autotopagnosia* *Anosognosia for hemiplegia* *Phantom limb* *Illusory additional limbs* *Somatoparaphrenia* Psychiatric e.g., *Depersonalisation*	As in Schilder	

Evaluating the impact of the concepts of body schema and of body image on modern models is difficult, since the two terms have often been used interchangeably, for instance by Gerstmann and Lhermitte. Conrad (1933) criticised the term body schema, used as a synonymous of image, as conceptually undefined. This vagueness inevitably led to a great deal of confusion. Similarly, Critchley (1979, p. 93) wrote that ‘Obviously this chaotic state of affairs is untenable’. Conrad (1933) suggested that the disorders of the body schema, such as anosognosia and autotopagnosia, are primarily disorders of consciousness. Critchley's views (1979, p. 92) were broadly similar, with corporeal awareness being primarily based on the body schema/image. More recently, Berlucchi and Aglioti (1997) suggested that the inconsistence and variability in the definition of the body schema/image, and the different use of the two terms made by different authors, make a good case for completely giving them up.

Conversely, some accounts maintain the putative bipartite distinction between body schema and body image, with reference to the dichotomy between the two visual systems (Ungerleider & Haxby, 1994), namely the ‘what’ (perceptual, image), and the ‘where’ (spatial, schema), the ‘visual system for action’ and the ‘visual system for perception’ (Milner & Goodale, 2006). Paillard (1999) suggests that, in the body, the schema corresponds to the ‘where’ system, and the image to the ‘what’ system. This conclusion was based on a double dissociation (Vallar, 2000). Patient R. S., with a central deafferentation, due to an obstruction in the left posterior parietal artery, resulting in a lesion of the posterior parietal lobe, showed a deficit in the detection of tactile stimuli, associated to an above chance localisation, a ‘blind touch’ phenomenon, analogous to ‘blindsight’ (Danckert et al., 2021). Another patient, G.L., with a peripheral deafferentation, due to a polyneuropathy, selectively affecting the large myelinated sensory fibres, and an intact motor system, showed a selective deficit of the localisation by pointing of cold stimuli, only when blindfolded (Paillard, 1999). G.L. was however capable to localise (verbally or by pointing on a body picture) consciously detected tactile stimuli. These patterns were interpreted as a deficit of the body image in R.S., and of the body schema in G.L. (see also Dijkerman & de Haan, 2007). A similar perspective characterises the body schemata, with reference to Head and Holmes' (1911) postural schema, as unconscious, referring to an on‐line, real‐time plastic, representation of one's own body in space, derived mainly from proprioceptive inputs, and interacting with motor systems for action. The body images, by contrast, are conscious representations, including conceptual knowledge about the body (Holmes & Spence, 2004). Gallagher and Cole's (1995) patient I.W. had suffered from an acute sensory neuropathy, in which large fibres below the neck had been destroyed. I.W. had a complete loss of proprioceptive function and sense of touch below the neck. ‘To maintain his posture and to control his movement I.W. must not only keep parts of his body in his visual field, but also conceptualize postures and movements’. On the basis of this dissociation, Gallagher and Cole (1995) concluded that I.W.'s unconscious postural body schema was damaged, and that his control over posture and movement was achieved through a partial compensation by a perceptual, conscious, body image.

Schwoebel and Coslett (2005) suggest a tripartite distinction: (i) ‘on‐line sensorimotor representations’, involved in several levels of motor control (i.e., the plastic postural schema of Head & Holmes, 1911); (ii) a ‘structural description’, conceived as a topological map of the body and of body parts, consistent with Pick's (1922) suggestion, and defective in autotopagnosia; (iii) the ‘body image’, representing semantic and lexical information about the human body (body parts' names, associations between body parts and artefacts, functions of body parts). Somewhat similarly, although with a differently organised and more complex taxonomy, Longo et al. (2010) distinguish the peripheral processes of ‘somatosensation’ from two groups of central internal representations of the body. ‘Somatoperception’ includes Head and Holmes' (1911) postural (unconscious) and superficial (conscious) schemata, Bonnier's (1905) conscious schema, which build up a conscious body image, and ‘emotion‐in‐body’ (i.e., affective processing of and responses to somatic stimuli), likely related also to visceral sensations (see Deny & Camus, 1905). ‘Somatorepresentation’, based on Pick's (1922) views, comprises structural/topological knowledge of one's own body, and knowledge about the arrangement of body parts, encyclopaedic and lexical‐semantic knowledge about bodies, and ‘emotion‐about‐body’ (i.e., formation of attitudes towards the body).

As noted above, dichotomies such as perception vs. action and conscious vs. nonconscious processes have been used to describe the differences between the image(s) and the schema(ta) of the body. However, the boundaries between the two types of bodily representations are fuzzy, as also suggested by their frequent utilisation as interchangeable terms (Critchley, 1979, p. 93). For instance, in Longo et al.'s (2010) model, somatoperception includes both conscious and unconscious processes, and some components also involved in action (i.e., the unconscious postural schema). As an attempt to overcome these problems, Moseley et al. (2012) suggested that a neural network of multisensory and homeostatic brain areas may be responsible for maintaining a ‘body‐matrix’, a dynamic neural representation that integrates body‐centred spatial sensory data, and also extends beyond the body surface to integrate both somatotopic and peri‐personal sensory data: an ‘extended body schema’, as suggested by Head and Holmes (1911), to include noncorporeal objects that bear a systematic relation to the body itself, such as clothes, ornaments and tools (Berlucchi & Aglioti, 1997). The matrix articulates into several components (somatotopic, peri‐personal and body‐centred spatial representations), localised in different cortical regions and an autonomic regulation system (see Deny & Camus, 1905, for an early suggestion of a contribution of ‘visceral’, internal, sensations to the representation of the body) in the brain stem. Notably, Moseley et al.'s (2012) ‘body‐matrix’, does not include the lexical‐semantic and encyclopaedic components of bodily representations.

Multisensory integration has been considered most relevant in the building up of the body schema/image, conceived as conscious/aware representations of the body (Conrad, 1933; Critchley, 1979; de Vignemont, 2010). The precise mechanisms of such an integration were however unclear, ranging from a summative complex of the different sensory inputs to a *Gestalt* (see Conrad, 1933, for a critical discussion of Schilder's, 1935, account). This vagueness was likely to be due to the lack of experimental data, since such hypotheses were primarily based on non‐quantitative clinical observations. In recent years, experimental studies of bodily illusions have provided some insight into the mechanisms of crossmodal integration of inputs from different sensory modalities into an internal representation of the body (see Moseley et al., 2012 for review). For instance, in the rubber hand illusion, the participant's hand is placed out of view and the artificial, rubber hand is placed in view, and synchronous strokes are delivered to both hands. In less than a minute, most participants report that they can feel the touch on the rubber hand and begin to feel a sense of ownership over it. This illusion is reminiscent of the deficit of the feeling of ownership of body parts, typically the hand, found in somatoparaphrenia (Vallar & Ronchi, 2009). Since asynchronous stroking rarely elicits the illusion, the strength of the illusion depends on the crossmodal congruence between what participants feel via the somatosensory pathways and what they see (Tsakiris, 2010).

In sum, the concepts ‘body schema’ and ‘body image’ have provided explanatory models for several bodily disorders, ranging from neuropsychological to psychiatric deficits, as anorexia nervosa, bulimia nervosa, conversion disorder (hysteria), depersonalisation, hypochondria, Alice in Wonderland syndrome (distorted awareness of the size, mass, shape of the body or of its position in space, autoscopic hallucinations, Fine, 2013; Todd, 1955) and Out of the Body Experience (disembodiment: location of the self outside one's own body; autoscopy, Blanke et al., 2004; Bünning & Blanke, 2005). This list, amounting to over 40 disorders (de Vignemont, 2010), implies that multiple internal representations of the body exist. Starting from a bipartite representation (schema and image), a fractionation process has occurred, as in many domains of neuropsychology (Vallar, 2000), unveiling the multicomponent nature of the functionally different internal representations of the body, based on different neural networks (Berlucchi & Aglioti, 1997; Moseley et al., 2012). Perhaps, the more neutral term ‘representation’ of the body might be used instead of the controversial body ‘schema’ and ‘image’.

## AUTHOR CONTRIBUTIONS

**Giuseppe Vallar:** Conceptualization; investigation; funding acquisition; writing – original draft; methodology; validation; visualization; writing – review and editing; software; formal analysis; project administration; data curation; supervision; resources.

## CONFLICT OF INTEREST STATEMENT

The author has no conflict of interest for this article.

## Data Availability

Data sharing is not applicable to this article as no new data were created or analyzed in this study.

## References

[jnp12389-bib-0001] Adair, J. C. , Na, D. L. , Schwartz, R. L. , Fennell, E. M. , Gilmore, R. L. , & Heilman, K. M. (1995). Anosognosia for hemiplegia: Test of the personal neglect hypothesis. Neurology, 45(12), 2195–2199. 10.1212/wnl.45.12.2195 8848192

[jnp12389-bib-0002] Berlucchi, G. , & Aglioti, S. M. (1997). The body in the brain: Neural bases of corporeal awareness. Trends in Neurosciences, 20(12), 560–564. 10.1016/s0166-2236(97)01136-3 9416668

[jnp12389-bib-0003] Berlucchi, G. , & Aglioti, S. M. (2010). The body in the brain revisited. Experimental Brain Research, 200(1), 25–35. 10.1007/s00221-009-1970-7 19690846

[jnp12389-bib-0004] Berthier, M. , Starkstein, S. , & Leiguarda, R. (1988). Asymbolia for pain: A sensory‐limbic disconnection syndrome. Annals of Neurology, 24(1), 41–49. 10.1002/ana.410240109 3415199

[jnp12389-bib-0005] Berti, A. M. , Bottini, G. , Gandola, M. , Pia, L. , Smania, N. , Stracciari, A. , Castiglioni, I. , Vallar, G. , & Paulesu, E. (2005). Shared cortical anatomy for motor awareness and motor control. Science, 309(5733), 488–491. 10.1126/science.1110625 16020740

[jnp12389-bib-0006] Bisiach, E. , Perani, D. , Vallar, G. , & Berti, A. (1986). Unilateral neglect: Personal and extra‐personal. Neuropsychologia, 24(6), 759–767. 10.1016/0028-3932(86)90075-8 3100983

[jnp12389-bib-0007] Bisiach, E. , Rusconi, M. L. , & Vallar, G. (1991). Remission of somatoparaphrenic delusion through vestibular stimulation. Neuropsychologia, 29(10), 1029–1031. 10.1016/0028-3932(91)90066-H 1762671

[jnp12389-bib-0008] Bisiach, E. , Vallar, G. , Perani, D. , Papagno, C. , & Berti, A. (1986). Unawareness of disease following lesions of the right hemisphere: Anosognosia for hemiplegia and anosognosia for hemianopia. Neuropsychologia, 24(4), 471–482. 10.1016/0028-3932(86)90092-8 3774133

[jnp12389-bib-0087] Blanke, O. , Landis, T. , Spinelli, L. , & Seeck, M. (2004). Out‐of‐body experience and autoscopy of neurological origin. Brain, 127(2), 243–258. 10.1093/brain/awh040 14662516

[jnp12389-bib-0009] Bonnier, P. (1905). L'aschématie. Revue Neurologique, 13, 605–609.

[jnp12389-bib-0010] Bottini, G. , Sterzi, R. , & Vallar, G. (1992). Directional hypokinesia in spatial hemineglect: A case study. Journal of Neurology, Neurosurgery and Psychiatry, 55(7), 562–565. 10.1136/jnnp.55.7.562 1640231 PMC489166

[jnp12389-bib-0011] Brain, W. R. (1941). Visual disorientation with special reference to lesions of the right cerebral hemisphere. Brain, 64(4), 244–272. 10.1093/brain/64.4.244 PMC1998360 19992404

[jnp12389-bib-0012] Brugger, P. , & Lenggenhager, B. (2014). The bodily self and its disorders: Neurological, psychological and social aspects. Current Opinion in Neurology, 27(6), 644–652. 10.1097/WCO.0000000000000151 25333602

[jnp12389-bib-0088] Bünning, S. , & Blanke, O. (2005). The out‐of body experience: Precipitating factors and neural correlates. Progress in Brain Research, 150(24), 331–352. 10.1016/S0079-6123(05)50024-4 16186034

[jnp12389-bib-0013] Cappa, S. , Sterzi, R. , Vallar, G. , & Bisiach, E. (1987). Remission of hemineglect and anosognosia during vestibular stimulation. Neuropsychologia, 25(5), 775–782. 10.1016/0028-3932(87)90115-1 3501552

[jnp12389-bib-0014] Charcot, J. M. (1879). Lectures on the diseases of the nervous system: Delivered at la Salpêtrière (translated from the second edition by George Sigerson). Henry C Lea.

[jnp12389-bib-0015] Committeri, G. , Piervincenzi, C. , & Pizzamiglio, L. (2018). Personal neglect: A comprehensive theoretical and anatomo–clinical review. Neuropsychology, 32(3), 269–279. 10.1037/neu0000409 29620402

[jnp12389-bib-0016] Conrad, K. (1933). Das Korperschema Eine kritische Studie und der Versuch einer Revision. Zeitschrift für Die Gesamte Neurologie Und Psychiatrie, 147(1), 346–369. 10.1007/BF02870452

[jnp12389-bib-0017] Critchley, M. (1953). The parietal lobes. Hafner.

[jnp12389-bib-0018] Critchley, M. (1979). The divine banquet of the brain and other essays. Raven Press.

[jnp12389-bib-0019] Danckert, J. , Striemer, C. , & Rossetti, Y. (2021). Blindsight. In J. J. S. Barton & A Leff (eds.), Handbook of Clinical Neurology, Neurology of Vision and Visual Disorders. (Vol. 178, pp.297–310. 10.1016/B978-0-12-821377-3.00016-7 33832682

[jnp12389-bib-0020] De Renzi, E. , & Scotti, G. (1970). Autotopagnosia: Fiction or reality?: Report of a case. Archives of Neurology, 23(3), 221–227. 10.1001/archneur.1970.00480270031005 5456719

[jnp12389-bib-0021] de Vignemont, F. (2010). Body schema and body image—Pros and cons. Neuropsychologia, 48(3), 669–680. 10.1016/j.neuropsychologia.2009.09.022 19786038

[jnp12389-bib-0022] Denburg, N. , & Tranel, D. (2003). Acalculia and disturbances of the body schema. In K M. Heilman & E. Valenstein (Eds.), Clinical neuropsychology (4th ed., pp. 161–194). Oxford University Press.

[jnp12389-bib-0023] Denes, G. (1989). Disorders of body awareness and body knowledge. In F. Boller & J. Grafman (Eds.), Handbook of Neuropsychology (Vol. 2, pp. 207–228). Elsevier.

[jnp12389-bib-0024] Dening, T. R. , & Berrios, G. E. (1994). Autoscopic phenomena. The British Journal of Psychiatry, 165(6), 808–817. 10.1192/bjp.165.6.808 7881784

[jnp12389-bib-0025] Deny, G. , & Camus, P. (1905). Sur une forme d'hypocondrie aberrante due a la perte de la coscience du corps. Revue Neurologique (Paris), 13(9), 460–467.

[jnp12389-bib-0026] Dieguez, S. (2018). Cotard syndrome. Frontiers of Neurology and Neuroscience, 42, 23–34. 10.1159/000475679 29151088

[jnp12389-bib-0027] Dijkerman, H. C. , & de Haan, E. H. F. (2007). Somatosensory processes subserving perception and action. Behavioral and Brain Sciences, 30(2), 189–201. 10.1017/S0140525X07001392 17705910

[jnp12389-bib-0028] Ehrenwald, H. (1931). Anosognosie und Depersonalisation. Nervenarzt, 4, 681–688.

[jnp12389-bib-0029] Fine, E. J. (2013). The Alice in wonderland syndrome. Progress in Brain Research, 206(8), 143–156. 10.1016/B978-0-444-63364-4.00025-9 24290480

[jnp12389-bib-0030] Foerster, O. (1903). Ein Fall von elementärer allgemeiner Somatopsychose. (Afunktion der Somatopsyche). Ein Beitrag zur Frage der Bedeutung der Somatopsyche für das Wahrnehmungsvermögen. Monatsschrift für Psychiatrie und Neurologie, 14, 189–205.

[jnp12389-bib-0031] Frederiks, J. A. M. (1985). Disorders of the body schema. In P. J. Vinken , G. W. Bruyn , H. L. Klawans , & J. A. M. Frederiks (Eds.), Handbook of clinical neurology. Clinical neuropsychology. Revised series 1 (Vol. 45, pp. 373–393). Elsevier.

[jnp12389-bib-0032] Gallagher, S. , & Cole, J. (1995). Body image and body schema in a deafferented subject. The Journal of Mind and Behavior, 16(4), 369–389. https://www.jstor.org/stable/43853796

[jnp12389-bib-0033] Gandola, M. , Invernizzi, P. , Sedda, A. , Ferrè, E. R. , Sterzi, R. , Sberna, M. , Paulesu, E. , & Bottini, G. (2012). An anatomical account of somatoparaphrenia. Cortex, 48(9), 1165–1178. 10.1016/j.cortex.2011.06.012 21774922

[jnp12389-bib-0034] Gerstmann, J. (1940). Syndrome of finger agnosia, disorientation for the right and left, agraphia and acalculia. Archives of Neurology and Psychiatry, 44(2), 398–408. 10.1001/archneurpsyc.1940.02280080158009

[jnp12389-bib-0035] Halligan, P. W. , Marshall, J. C. , & Wade, D. T. (1995). Unilateral somatoparaphrenia after right hemisphere stroke: A case description. Cortex, 31(1), 173–182. 10.1016/s0010-9452(13)80115-3 7781314

[jnp12389-bib-0036] Head, H. , & Holmes, G. (1911). Sensory disturbances from cerebral lesions. Brain, 34(2–3), 102–254. 10.1093/brain/34.2-3.102

[jnp12389-bib-0037] Hécaen, H. (1972). Introduction à la neuropsychologie. Larousse.

[jnp12389-bib-0038] Hécaen, H. , & De Ajuriaguerra, J. (1952). Méconnaissances et hallucinations corporelles: Intégration et désintégration de la somatognosie. Masson. 13021133

[jnp12389-bib-0039] Heilman, K. M. , Watson, R. T. , & Valenstein, E. (2003). Neglect and related disorders. In K. M. Heilman & E. Valenstein (Eds.), Clinical Neuropsychology (4th ed., pp. 296–346). Oxford University Press.

[jnp12389-bib-0040] Holmes, N. P. , & Spence, C. (2004). The body schema and multisensory representation(s) of peripersonal space. Cognitive Processing, 5(2), 94–105. 10.1007/s10339-004-0013-3 16467906 PMC1350799

[jnp12389-bib-0041] Kleinschmidt, A. , & Rusconi, E. (2011). Gerstmann meets Geschwind: A crossing (or kissing) variant of a subcortical disconnection syndrome? The Neuroscientist, 17(6), 633–644. 10.1177/1073858411402093 21670425

[jnp12389-bib-0042] Lhermitte, J. (1939). L'image de notre corps. Éditions de la Nouvelle Revue Critique, Imprimerie Chantenay.

[jnp12389-bib-0043] Lhermitte, J. (1951). Visual hallucination of the self. British Medical Journal, 1(4704), 431–434. 10.1136/bmj.1.4704.431 14821434 PMC2068415

[jnp12389-bib-0044] Lhermitte, J. , & Tchehrazi, E. (1937). L'image du moi corporel et ses déformations pathologiques. L'Encéphale, 32(1), 1–24.

[jnp12389-bib-0045] Limanowski, J. (2022). Precision control for a flexible body representation. Neuroscience & Biobehavioral Reviews, 134, 104401. 10.1016/j.neubiorev.2021.10.023 34736884

[jnp12389-bib-0046] Lippman, C. W. (1952). Certain hallucinations peculiar to migraine. The Journal of Nervous and Mental Disease, 116(4), 346–351. https://journals.lww.com/jonmd/citation/1952/10000/certain_hallucinations_peculiar_to_migraine.9.aspx 12991095 10.1097/00005053-195210000-00009

[jnp12389-bib-0047] Loetscher, T. , Regard, M. , & Brugger, P. (2006). Misoplegia: A review of the literature and a case without hemiplegia. Journal of Neurology, Neurosurgery, and Psychiatry, 77(9), 1099–1100. 10.1136/jnnp.2005.087163 16914766 PMC2077726

[jnp12389-bib-0048] Longo, M. R. , Azañón, E. , & Haggard, P. (2010). More than skin deep: Body representation beyond primary somatosensory cortex. Neuropsychologia, 48(3), 655–668. 10.1016/j.neuropsychologia.2009.08.022 19720070

[jnp12389-bib-0049] Maravita, A. (2006). From ‘body in the brain’ to ‘body in space’. Sensory and intentional components of body representation. In G. Knoblich , I. Thornton , M. Grosjean , & M. Shiffrar (Eds.), Human Body Perception from the Inside Out (pp. 65–88). Oxford University Press.

[jnp12389-bib-0050] Milner, A. D. , & Goodale, M. A. (2006). The Visual Brain in Action (2nd ed.). Oxford University Press.

[jnp12389-bib-0051] Moro, V. , Pernigo, S. , Tsakiris, M. , Avesani, R. , Edelstyn, N. M. J. , Jenkinson, P. M. , & Fotopoulou, A. (2016). Motor versus body awareness: Voxel‐based lesion analysis in anosognosia for hemiplegia and somatoparaphrenia following right hemisphere stroke. Cortex, 83, 62–77. 10.1016/j.cortex.2016.07.001 27494375

[jnp12389-bib-0052] Moseley, G. L. , Gallace, A. , & Spence, C. (2012). Bodily illusions in health and disease: Physiological and clinical perspectives and the concept of a cortical ‘body matrix’. Neuroscience and Biobehavioral Reviews, 36(1), 34–46. 10.1016/j.neubiorev.2011.03.013 21477616

[jnp12389-bib-0053] Munk, H. (1890). Über die Functionen der Grosshirnrinde (2nd ed.). Hirshwald.

[jnp12389-bib-0054] Nightingale, S. (1982). Somatoparaphrenia: A case report. Cortex, 18(3), 463–467. 10.1016/S0010-9452(82)80043-9 7151454

[jnp12389-bib-0055] Ogden, J. A. (1985). Autotopagnosia. Occurrence in a patient without nominal aphasia and with an intact ability to point to parts of animals and objects. Brain, 108(4), 1009–1022. 10.1093/brain/108.4.1009 4075073

[jnp12389-bib-0056] Paillard, J. (1999). Body schema and body image. A double dissociation in deafferented patients. In G. N. Gantchev , S. Mori , & J. Massion (Eds.), Motor Control, Today and Tomorrow (pp. 197–214). Academic Publishing House.

[jnp12389-bib-0057] Park, H.‐D. , & Blanke, O. (2019). Coupling inner and outer body for self‐consciousness. Trends in Cognitive Sciences, 23(5), 377–388. 10.1016/j.tics.2019.02.002 30826212

[jnp12389-bib-0058] Pia, L. , Neppi‐Modona, M. , Ricci, R. , & Berti, A. (2004). The anatomy of anosognosia for hemiplegia: A meta‐analysis. Cortex, 40(2), 367–377. 10.1016/s0010-9452(08)70131-x 15156794

[jnp12389-bib-0059] Pick, A. (1922). Störung der Orientierung am eigenen Körper. Beitrag zur Lehre vom Bewusstsein des eigenen Körpers. Psychologische Forschung, 1, 303–318.

[jnp12389-bib-0060] Podoll, K. , & Robinson, D. (2000). Macrosomatognosia and microsomatognosia in migraine art. Acta Neurologica Scandinavica, 101(6), 413–416. 10.1034/j.1600-0404.2000.9s334.x 10877160

[jnp12389-bib-0061] Poeck, K. , & Orgass, B. (1971). The concept of the body schema: A critical review and some experimental results. Cortex, 7(3), 254–277. 10.1016/s0010-9452(71)80005-9 4946208

[jnp12389-bib-0062] Rode, G. , Vallar, G. , Revol, P. , Tilikete, C. , Jacquin‐Courtois, S. , Rossetti, Y. , & Farnè, A. (2012). Facial macrosomatognosia and pain in a case of Wallenberg's syndrome: Selective effects of vestibular and transcutaneous stimulations. Neuropsychologia, 50(2), 245–253. 10.1016/j.neuropsychologia.2011.11.018 22142667

[jnp12389-bib-0063] Ronchi, R. , Park, H.‐D. , & Blanke, O. (2018). Bodily self‐consciousness and its disorders. In G. Vallar & H. B. Coslett (Eds.), Handbook of Clinical Neurology. The Parietal Lobe. (Vol. 151, pp. 313–330). Elsevier. 10.1016/B978-0-444-63622-5.00015-2 29519466

[jnp12389-bib-0064] Roth, M. (1949). Disorders of the body image caused by lesions of the right parietal lobe. Brain, 72(1), 89–111. 10.1093/brain/72.1.89 18145438

[jnp12389-bib-0065] Rusconi, E. (2018). Gerstmann's syndrome: Historical and current perspectives. In G. Vallar & H. B. Coslett (Eds.), Handbook of Clinical Neurology. The parietal lobe. (Vol. 151, pp. 395–411). Elsevier. 10.1016/B978-0-444-63622-5.00020-6 29519471

[jnp12389-bib-0066] Salami, A. , Andreu‐Perez, J. , & Gillmeister, H. (2020). Symptoms of depersonalisation/derealisation disorder as measured by brain electrical activity: A systematic review. Neuroscience & Biobehavioral Reviews, 118, 524–537. 10.1016/j.neubiorev.2020.08.011 32846163

[jnp12389-bib-0067] Salanova, V. , Andermann, F. , Rasmussen, T. , Olivier, A. , & Quesney, L. F. (1995). Parietal lobe epilepsy Clinical manifestations and outcome in 82 patients treated surgically between 1929 and 1988. Brain, 118(3), 607–627. 10.1093/brain/118.3.607 7600082

[jnp12389-bib-0068] Schilder, P. (1923). Das Körperschema: Ein Beitrag zur Lehre vom Bewußtsein Des Eigenen Körpers. Springer‐Verlag.

[jnp12389-bib-0069] Schilder, P. (1935). The image and appearance of the human body. International Universities Press, Inc.

[jnp12389-bib-0070] Schwoebel, J. , & Coslett, H. B. (2005). Evidence for multiple, distinct representations of the human body. Journal of Cognitive Neuroscience, 17(4), 543–553. 10.1162/0898929053467587 15829076

[jnp12389-bib-0071] Semenza, C. , & Delazer, M. (2003). Pick's cases on body representation (1908, 1915, 1922): A retrospective assessment. In C. Code , C. W. Wallesch , Y. Joanette , & A. R. Lecour (Eds.), Classic cases in neuropsychology (Vol. 2, pp. 223–240). Psychology Press.

[jnp12389-bib-0072] Sierra, M. , & Berrios, G. E. (1997). Depersonalization: A conceptual history. History of Psychiatry, 8(30), 213–229. 10.1177/0957154X9700803002 11619439

[jnp12389-bib-0073] Sirigu, A. , Grafman, J. , Bressel, K. , & Sunderland, T. (1991). Multiple representations contribute to body knowledge processing. Evidence from a case of autotopagnosia. Brain, 114(1), 629–642. 10.1093/brain/114.1.629 2004260

[jnp12389-bib-0074] Stone, J. , & Vermeulen, M. (2016). Functional sensory symptoms. In M. Hallett , J. Stone , & A. Carson (Eds.), Handbook of clinical neurology. Functional Neurologic Disorders. (Vol. 139, pp. 271–281). Elsevier. 10.1016/B978-0-12-801772-2.00024-2 27719847

[jnp12389-bib-0075] Todd, J. (1955). The syndrome of Alice in wonderland. Canadian Medical Association Journal, 73(9), 701–704. 13304769 PMC1826192

[jnp12389-bib-0076] Tsakiris, M. (2010). My body in the brain: A neurocognitive model of body‐ownership. Neuropsychologia, 48(3), 703–712. 10.1016/j.neuropsychologia.2009.09.034 19819247

[jnp12389-bib-0089] Ungerleider, L. G. , & Haxby, J. V. (1994). ‘What’ and ‘where’ in the human brain. Current Opinion in Neurobiology, 4(2), 157–165. 10.1016/0959-4388(94)90066-3 8038571

[jnp12389-bib-0078] Vallar, G. (2000). The methodological foundations of human neuropsychology: Studies in brain‐damaged patients. In F. Boller , J. Grafman , & G. Rizzolatti (Eds.), Handbook of Neuropsychology (Vol. 1, pp. 305–344). Elsevier.

[jnp12389-bib-0079] Vallar, G. , Albini, F. , & Pisoni, A. (2024). Unilateral spatial neglect. In Reference Module in Neuroscience and Biobehavioral Psychology (pp. 1–24). Elsevier. 10.1016/B978-0-12-820480-1.00053-X

[jnp12389-bib-0080] Vallar, G. , & Rode, G. (2009). Commentary on Bonnier P. L'aschématie. *Revue Neurologique (Paris)* 1905;13: 605–9. Epilepsy and Behavior, 16(3), 397–400. 10.1016/j.yebeh.2009.09.001 19854110

[jnp12389-bib-0081] Vallar, G. , & Ronchi, R. (2009). Somatoparaphrenia: A body delusion. A review of the neuropsychological literature. Experimental Brain Research, 192(3), 533–551. 10.1007/s00221-008-1562-y 18813916

[jnp12389-bib-0082] Veronelli, L. , & Vallar, G. (2024). Left‐ and right‐side unilateral spatial neglect. Hemispheric differences. In C. Papagno & P. Corballis (Eds.), Cerebral Asymmetries. Elsevier Inc. 10.1016/B978-0-443-15646-5.00025-7 40074392

[jnp12389-bib-0083] Vocat, R. , Staub, F. , Stroppini, T. , & Vuilleumier, P. (2010). Anosognosia for hemiplegia: A clinical‐anatomical prospective study. Brain, 133(12), 3578–3597. 10.1093/brain/awq297 21126995

[jnp12389-bib-0084] Wernicke, C. (1906). Grundriss der Psychiatrie in klinischen Vorlesungen. Verlag von Georg Thieme.

[jnp12389-bib-0085] Yamazaki, K. , Hirata, K. , Mimuro, I. , & Kaitoh, Y. (2001). A case of dressing apraxia: contributory factor to dressing apraxia. Journal of Neurology, 248(3), 235–236. 10.1007/s004150170234 11355161

[jnp12389-bib-0086] Yeo, B. T. T. , Krienen, F. M. , Eickhoff, S. B. , Yaakub, S. N. , Fox, P. T. , Buckner, R. L. , Asplund, C. L. , & Chee, M. W. L. (2015). Functional specialization and flexibility in human association cortex. Cerebral Cortex, 25(10), 3654–3672. 10.1093/cercor/bhu217 25249407 PMC4598819

